# Transgenic Mouse Bioassay: Evidence That Rabbits Are Susceptible to a Variety of Prion Isolates

**DOI:** 10.1371/journal.ppat.1004977

**Published:** 2015-08-06

**Authors:** Enric Vidal, Natalia Fernández-Borges, Belén Pintado, Hasier Eraña, Montserrat Ordóñez, Mercedes Márquez, Francesca Chianini, Dolors Fondevila, Manuel A. Sánchez-Martín, Olivier Andreoletti, Mark P. Dagleish, Martí Pumarola, Joaquín Castilla

**Affiliations:** 1 IRTA, Centre de Recerca en Sanitat Animal (CReSA, IRTA-UAB), Campus de la Universitat Autònoma de Barcelona, Bellaterra, Spain; 2 CIC bioGUNE, Parque tecnológico de Bizkaia, Derio, Bizkaia, Spain; 3 Centro Nacional de Biotecnología (CNB), Campus de Cantoblanco, Cantoblanco, Madrid, Spain; 4 Department of Animal Medicine and Surgery, Veterinary faculty, Universitat Autònoma de Barcelona (UAB), Bellaterra (Cerdanyola del Vallès), Barcelona, Catalonia, Spain; 5 Moredun Research Institute, Pentlands Science Park, Bush Loan, Penicuik, Near Edinburgh, Scotland, United Kingdom; 6 Unidad de Generación de OMGs, S.E.A. Department of Medicine, University of Salamanca, Salamanca, Spain; 7 Ecole Nationale du Veterinaire, Service de Pathologie du Bétail, Toulouse, France; 8 IKERBASQUE, Basque Foundation for Science, Bilbao, Bizkaia, Spain; Dartmouth Medical School, USA, UNITED STATES

## Abstract

Interspecies transmission of prions is a well-established phenomenon, both experimentally and under field conditions. Upon passage through new hosts, prion strains have proven their capacity to change their properties and this is a source of strain diversity which needs to be considered when assessing the potential risks associated with consumption of prion contaminated protein sources. Rabbits were considered for decades to be a prion resistant species until proven otherwise recently. To determine the extent of rabbit susceptibility to prions and to assess the effects of passage of different prion strains through this species a transgenic mouse model overexpressing rabbit PrP^C^ was developed (*TgRab*). Intracerebral challenges with prion strains originating from a variety of species including field isolates (ovine SSBP/1 scrapie, Nor98- scrapie; cattle BSE, BSE-L and cervid CWD), experimental murine strains (ME7 and RML) and experimentally obtained ruminant (sheepBSE) and rabbit (*de novo* NZW) strains were performed. On first passage *TgRab* were susceptible to the majority of prions (Cattle BSE, SheepBSE, BSE-L, *de novo* NZW, ME7 and RML) tested with the exception of SSBP/1 scrapie, CWD and Nor98 scrapie. Furthermore, *TgRab* were capable of propagating strain-specific features such as differences in incubation periods, histological brain lesions, abnormal prion (PrP^d^) deposition profiles and proteinase-K (PK) resistant western blotting band patterns. Our results confirm previous studies proving that rabbits are not resistant to prion infection and show for the first time that rabbits are susceptible to PrP^d^ originating in a number of other species. This should be taken into account when choosing protein sources to feed rabbits.

## Author Summary

Prions, the infectious agents responsible for causing mad cow disease, amongst other diseases, can transmit from one species to another. For example, Bovine Spongiform Encephalopathy can transmit to humans resulting in invariably fatal variant Creutzfeldt-Jakob Disease. We wanted to study the susceptibility of rabbits as, until recently, they were considered a prion resistant species. Once proven otherwise, we wanted to know which particular prions rabbits were susceptible to. With this aim, a transgenic mouse was designed expressing the rabbit prion protein gene instead of the corresponding mouse gene to model the transmission barrier between rabbits and other species. The resultant mice where challenged with several field prion isolates including classical and atypical strains of Bovine Spongiform Encephalopathy, sheep Scrapie and cervid Chronic Wasting disease. The transgenic mice were susceptible to classical and atypical Bovine Spongiform Encephalopathy prions and also to mouse-adapted Scrapie prions. This information must be taken into account when assessing the risk of using ruminant derived protein as a protein source to feed rabbits.

## Introduction

Prions are protein based, genome devoid, infectious agents causing Transmissible Spongiform Encephalopathies (TSEs), a group of diseases classified as transmissible protein misfolding disorders [1,2]. Prions show a remarkable ability for interspecies transmission. Initially, a species barrier was defined, but extensive field and experimental evidence has been published proving that interspecies prion transmission is not an isolated phenomenon [3–6]. Interspecies transmission of prions has resulted in the generation of significant prion strain diversity and its incidence has been documented worldwide [3,4,7–10].

The existence of prion diseases has been documented for centuries with the earliest reports of scrapie cases dating back to 1732 [11]. In the last seven decades prions were also reported in other animal species, usually in the form of outbreaks, which somehow involved human intervention. Namely classical bovine spongiform encephalopathy (BSE-C) [12], feline spongiform encephalopathy (FSE) [13] and transmissible mink encephalopathy (TME) [14]. Humans can also be included in the list of TSE susceptible species due to the Fore tribe from Papua New Guinea suffering from Kuru [15] or the relatively newly created variant Creutzfeldt-Jakob disease (vCJD)[3]. Cervidae is another family of animals currently affected by a, yet uncontrolled, epizooty: chronic wasting disease (CWD) [16]. Although classical animal prion disease strains, as opposed to the so called atypical prion disease strains [17–20], have been documented for at least three centuries [11], sporadic spontaneous generation of atypical prions has probably existed for as long as susceptible species have been present in large enough numbers for the spontaneous event to occur. Currently there is no evidence to suggest that any mammalian species cannot undergo a spontaneous disease-linked prion protein misfolding event [21] as long as there are sufficient numbers of individuals with the necessary lifespan.

Although the mechanisms of interspecies prion transmission remain unknown, *in vitro* and *in vivo* studies have shown that species particularly susceptible to certain prion strains can actually be resistant to others which originated in the same or different species [9,21–28]. The ability of prions to adapt to new species and even generate new strains with pathobiological properties different from the original one is not an isolated phenomenon [9,27,29,30]. Therefore new prion strains may arise with the ability to infect new species previously considered resistant.

Normal cellular prion protein (PrP^C^) is a host encoded protein, particularly abundant in nerve cells, which when misfolded is believed to acquire pathological properties leading to TSE neurodegenerative disease [1]. Several studies argue that certain species specific amino acid sequences of PrP^C^ may render some species less susceptible to TSE [31–33] due to them being less prone to misfolding. This, along with absence of experimental evidence or TSE field cases described, led to belief that dogs, horses and rabbits (leporids) were more resistant to prion infection than other mammalian species [34,35].

Leporids have been the most intensely studied, both *in vivo* and *in vitro*, of the presumed prion disease resistant species. This is probably because rabbits are consumed by humans and also due to their comparatively small size and long lifespan which facilitates their use as experimental animals. Our group has proved recently, in contrast with the last three decades of reports, that rabbits are susceptible to prion diseases. Using protein misfolding cyclic amplification (PMCA), inocula where generated *in vitro* which were infectious and transmissible in this species [23] and more recent studies have proven that rabbit PrP^C^ has a misfolding ability comparable to other species as BSE prions have been shown to retain their *in vivo* strain properties after misfolding rabbit PrP^C^ [36]. Houdebine’s group studied whether the genetic background of rabbits was responsible for their apparent prion resistance generated transgenic rabbits expressing ovine PrP^C^. Upon inoculation with scrapie, these rabbits succumbed to prion disease further proving that leporids are not resistant to prion disease [37].

In the present paper we report an extensive evaluation of the susceptibility of *TgRab* mice to a variety of prion strains by means of *in vivo* experiments. A transgenic murine model has been generated *ad hoc* for this purpose which overexpresses the leporid PRNP on a mouse Prnp-null background. This model, denoted *TgRab*, has already been shown to correlate well with the rabbit model [23]. Our results show the susceptibility of rabbits has been vastly underestimated previously and that they behave similarly to other species whose vulnerability and/or resistance to prion disease also varies depending upon the prion disease strain encountered.

## Results

### Generation of *TgRab*: an *in vivo* model to evaluate rabbit susceptibility to prion infection

Even though the actual rabbit model would be more suitable for this purpose there are several significant limitations (size, cage space in biocontainment conditions, lifespan, expression levels, and budget required) that are easily overcome by using a transgenic mouse model and such models have been of great use within the field of prion research. Based on our previous experience a new mouse line was generated by pronuclear injection of a construct consisting of the moPrP promoter and the rabbit PrP sequence. From a total of seven positive animals identified from the 83 pups obtained, five animal founders transmitted the transgene to their progeny. After backcrossing to a line that did not express endogenous PrP (STOCK-Prnptm2Edin), expression levels of the transgene were analyzed by western blot. Two out of five transgenic lines expressed PrP at higher levels than the endogenous gene. However, only hemizygous line 58 showed a consistent expression pattern of 5x-6x that of the endogenous rabbit prion protein level and 10x-12x that of the endogenous mouse prion protein level (S2 Fig). This line was selected for further studies. The low expressing lines were discarded since PrP^C^ expression levels were lower than those found in WT rabbits and this would predictably diminish their susceptibility to prions.

### Preliminary *in vitro* challenge to assess rabbit susceptibility

In previous experiments normal rabbit brain homogenate was seeded *in vitro* with different prion strains before applying serial automated PMCA (saPMCA) to determine the ability of rabbit PrP^C^ to be converted by different PrP^res^ conformations. The results of some of these experiments have been reported previously such as seeding with cattle BSE which generated BSE-RaPrP^res^ [36]. Additional prion isolates were included in the present work, which successfully misfolded rabbit PrP^C^ *in vitro* including SSBP/1 sheep scrapie, ME7 and RML murine adapted scrapie strains and CWD. The following rabbit adapted strains were generated respectively: SSBP/1-RaPrP^res^, ME7-RaPrP^res^, RML-RaPrP^res^ and CWD-RaPrP^res^ (S1 Fig) [23]. Spontaneously misfolded PrP^res^ was also obtained from unseeded normal rabbit brain homogenates and named *de novo* RaPrP^res^. This spontaneous strain has been demonstrated to be infectious to rabbits [23]. Despite saPMCA not being a quantitative method, rabbit PrP^C^ appeared to be quite susceptible to misfolding since all seeds tested were able to generate PK-resistant RaPrP^res^ by or before round 7 and the unseeded homogenate produced RaPrP^res^ by round 13 [23]. All *in vitro*-derived RaPrP^res^ products were easily amplified further *in vitro*.

The western blotting migration pattern of the obtained RaPrP^res^, particularly the unglycosylated band, was similar to the strains of origin used in the bovine and ovine strains tested.

Accordingly, the following isolates were selected for *in vivo* challenge: BSE-C, SSBP/1, ME7, RML and CWD. Additionally, we included L-type atypical BSE (BSE-L) and Nor98 Atypical scrapie and the PMCA obtained *de novo* RaPrP^res^. The rationale for including the latter, *in vitro* generated, PrP^res^ was that it was able to infect the natural host i.e. rabbits, our species of study [23], *de novo* RaPrP^res^ was the only positive control available. Finally, *de novo* NZW prions (obtained from rabbits infected with *de novo* RaPrP^res^) were also inoculated into this transgenic mouse model and even though most of these results have already been published [23], some of them are discussed in the present paper.

### A valid model to study prion susceptibility and transmission in rabbits

**Fig 1:**
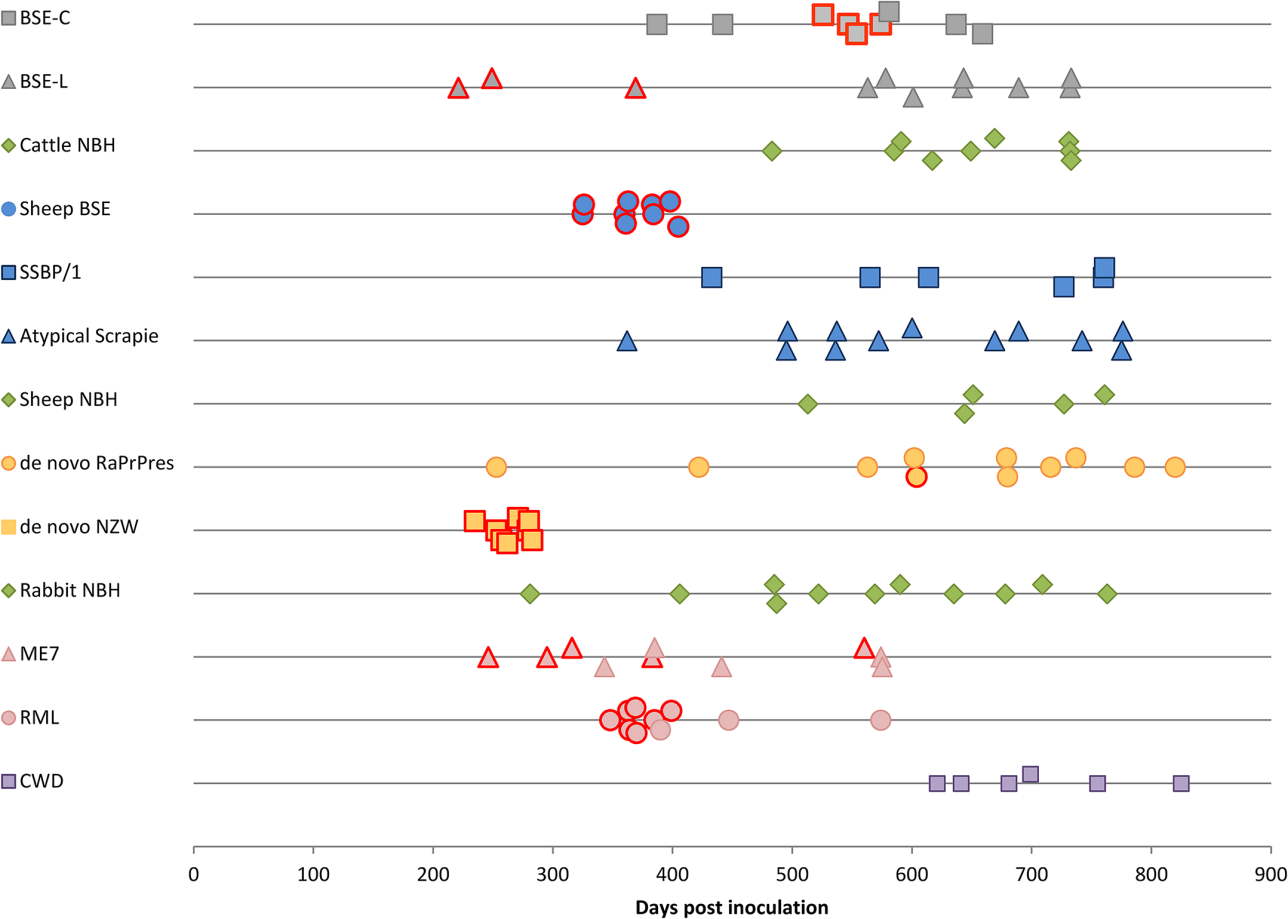
*TgRab* survival times after inoculation. Each dot corresponds to a mouse. Dots with a red margin represent TSE positive animals. Green dots are negative controls; grey dots correspond to cattle strains; blue dots to sheep strains; yellow dots to rabbit originating strains; pink dots are murine strains and purple dots to CWD strain.

As reported for rabbits showing very long incubation times (766 dpi) and a 33% attack rate (1/3) [23], *TgRab* mice were also susceptible to *de novo* RaPrP^res^ with a low attack rate (1/11) and a rather long incubation period of 604 dpi. However, upon inoculation with rabbit *in vivo*-adapted *de novo* RaPrP^res^ (*de novo* NZW) the *TgRab* mice developed a 100% attack rate (8/8) with a shortened incubation period of 256 (±5) dpi (Table 1 and Fig 1). The same rate was obtained by Chianini *et al*. [23] in rabbits inoculated (in second passage) with this prion strain (Table 1). *In vivo* experimental challenges in rabbits and *TgRab* mice have shown a good correlation making the transgenic mouse model overexpressing rabbit PrP^C^ a valid model to study rabbit prion susceptibility.

**Table 1:** Comparison of TgRab mice and actual rabbit bioassays.

	Animal model inoculated	PrP species of origin in inoculum	Strain origin	Attack rate	Survival time of positive animals (dpi) (±SEM)
*de novo* RaPrP^res^	tgRab	Rabbit	PMCA	**1**/11	604
*de novo*—NZW	tgRab	Rabbit	Experimental	**8**/8	265 (±5)
*de novo* RaPrP^res^	NZW Rabbit^a^	Rabbit	PMCA	**1**/3	766
*de novo*—NZW	NZW Rabbit	Rabbit	Experimental	**10**/10	612 (±7)

^a^ Result published in Chianini *et al*. 2012. NZW: New Zealand White.

### A model susceptible to bovine, ovine and murine prions

Even though rabbits had been considered resistant to prion infection until recently [23], *TgRab* mice could be infected with a number of the prions tested. Prions originating from BSE, i.e. cattle BSE-C and sheep BSE-C, were both infectious (Table 2). **Cattle BSE-C** showed an attack rate of 44.4% with an incubation period of 551(±10) dpi. Interestingly **Sheep BSE-C** showed a 100% attack rate and a significantly shortened incubation period of 368(±12) dpi (*P* = 0.0069, Mann-Whitney test) without previous adaptation to rabbit compared to cattle BSE-C (Fig 1). This supports, once again, the idea that after passage through sheep BSE-C shows enhanced virulence [29].

**Table 2:** Attack rates and survival times (±SEM) of the inoculated *TgRab* mice.

	PrP species of origin in inoculum	Strain origin	Attack rate^a^	Survival time of positive animals (dpi) (mean±SEM)
BSE-C	Cattle	Field isolate	**4**/9 (44%)	551 (±10)
Sheep BSE	Sheep	Experimental	**9**/9 (100%)	368 (±10)
BSE-L	Cattle	Field isolate	**3**/11(27%)	280 (±26)
SSBP/1	Sheep	Field isolate	0/6	775^b^
Atypical Scrapie	Sheep	Field isolate	0^c^/12	775^b^
CWD	Cervid	Field isolate	0/6	825^b^
ME7	Murine	Experimental	**5**/10 (50%)	360 (±41)
RML	Murine	Experimental	**7**/10 (70%)	371 (±6)
Cattle NBH	Cattle	Negative Control	0/9	810^b^
Rabbit NBH	Rabbit	Negative Control	0/11	760^b^
Sheep NBH	Sheep	Negative Control	0/5	761^b^

^a^Animals were considered TSE positive when spongiform lesions and/or PrP^d^ was detected, either through IHC, WB or ELISA. Inconclusive results were confirmed by using more than one PrP^d^ detection method. ND: Not determined, SEM: Standard Error of the Mean, NBH: Normal Brain Homogenate.

^b^All animals TSE negative. Age at death of the oldest animal of the group (spontaneous deaths or humanely euthanized due to non-TSE causes). Individual survival times are shown in Fig 1.

^c^One animal, euthanized at 742dpi, showed a positive result to PrP^d^ ELISA but negative to the remaining tests (WB, IHC, HP) a second passage is ongoing.

The picture with scrapie-originating prion isolates was quite different. **SSBP/1** prions were not able to infect *TgRab* (mice survived for longer than 750 dpi). Two other murine adapted classical scrapie prion sources were tested, ME7 and RML, and both strains readily infected *TgRab* mice with attack rates of 50% and 70% and incubation periods of 360(±41) and 371(±6) dpi, respectively (Fig 1). Therefore, prion strains originating from classical scrapie were transmissible to *TgRab* mice but only after being adapted previously to rodents. This situation is similar to that found with CWD which will infect hamsters readily after passage through ferrets [9].

### Challenge with atypical ovine and bovine prions

The new *TgRab* model was further characterized by testing its susceptibility to atypical prion strains using the more frequent isolates for each species, BASE (BSE-L, cattle) and Nor89 (sheep). *TgRab* mice were resistant to infection on first passage with atypical scrapie prions (living up to 775 dpi) (Fig 1) with one exception: a single animal (euthanized at 742 dpi) showed a positive result for PrP^d^ by ELISA but was negative when examined by western blotting and IHC and showed no TSE related spongiform change. A second passage is ongoing to determine if this animal was truly infected.

A 27% attack rate was present in the group inoculated with BSE-L with a mean incubation period of 280(±26) dpi, a similar rate to that of cattle BSE-C but with a much shorter incubation period (the number of positive animals per group was too low to assess statistical significance).

### *TgRab* are not susceptible to CWD on first passage

As mentioned before, *in vitro* adaptation of CWD prion to rabbit PrP^c^ was successful which indicated a potential susceptibility of rabbits *in vivo*. However, *TgRab* mice inoculated with CWD did not show any indication of a TSE on first passage, living up to 825 dpi (Fig 1 and Table 2). A second passage is ongoing to confirm these results.

### *TgRab* mice: a model able to propagate distinct prion strains

Biochemical and neuropathological characterization of the brains of the inoculated mice strongly suggests that *TgRab* mice are not only susceptible to multiple prion strains but are also able to maintain their strain features.

**Fig 2:**
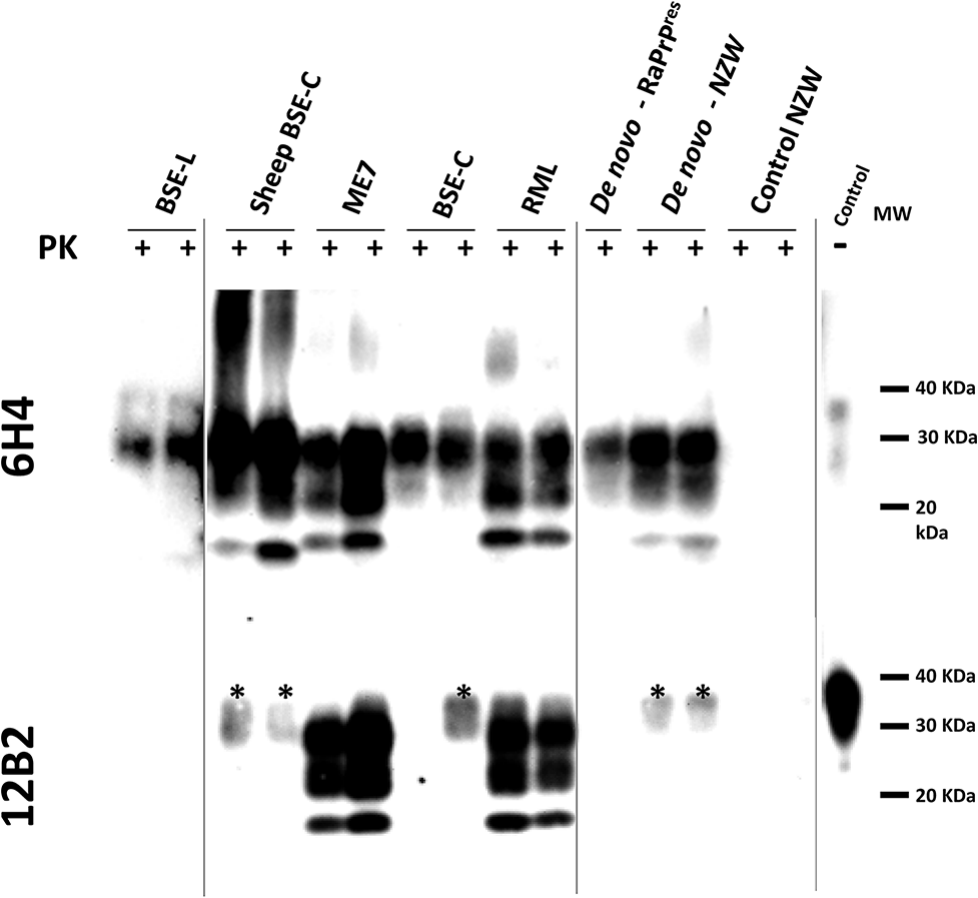
Biochemical analysis of brain homogenates from *TgRab* mice inoculated with different prion strains. Two representative brain homogenates (per group) from *TgRab* mice inoculated with different prion strains [cattle: BSE-L and BSE-C; mouse: RML and ME7; sheep: SSPB/1, sheep BSE-C and atypical scrapie; deer: CWD; rabbit: *de novo*–RaPrP^res^ (*in vitro* sample) and *de novo*–NZW (*in vivo* sample)] were digested with 100 μg/ml of proteinase K (PK) and analyzed by western blot using two different monoclonal antibodies (upper blot- 6H4 and lower blot– 12B2). Differential electrophoretic migrations and glycosylation patterns observed are consistent with the origin of the prion strains used for inoculation. Control NZW: Normal rabbit brain homogenate. MW: Molecular weight. Vertical lines separate blots with different exposition times.

Western blotting analysis of *TgRab* brain homogenates after protease K digestion revealed the characteristic three-band pattern with a predominant diglycosylated band and a 19-20kDa unglycosylated band in mice inoculated with BSE-derived strains (Fig 2). The brains of mice inoculated with RML showed a typical predominance of the monoglycosylated band and a 21kDa unglycosylated band. As shown in the 12B2 antibody developed membrane only ME7 and RML inoculated mice fully maintained the N-terminus specific epitope after PK digestion (Fig 2). The migration pattern of the bands from mice inoculated with *de novo* strains, both with the *in vitro* generated (*de novo*-RaPrP^res^) and the one obtained from NZW rabbits (*de novo* NZW), was constant and showed a similar pattern to BSE-C even though, as shown later, the immunohistochemical features differed completely. No bands were observed in western blots of brains of mice inoculated with SSBP/1, Atypical scrapie nor CWD or in any of the negative controls.

**Fig 3:**
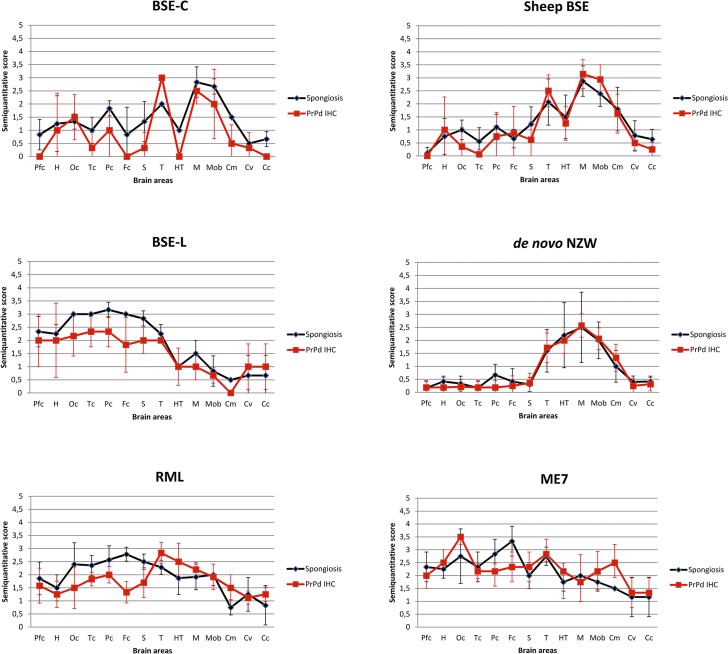
Brain lesion and PrP^d^ deposit distribution of the first passage of several prion strains in *TgRab* mice. Brain lesion profiles and PrP^d^ deposition profiles represent the mean semi-quantitative scoring (0–4, vertical axis) of the spongiform lesions (black) and the immunohistochemical labelling of PrP^d^ deposits (red) against 14 brain regions (Pfc: piriform cortex, H: hippocampus, Oc: occipital cortex, Tc: temporal cortex, Pc: parietal cortex, Fc: frontal cortex, S: striatum, T: thalamus, HT: hypothalamus, M: mesencephalon, Mob: medulla oblongata, Cm: cerebellar nuclei, Cv: cerebellar vermis, Cc: cerebellar cortex). Note that classical BSE-originating strains (BSE-C and Sheep BSE) generate very similar shaped brain profiles with low scores in the hypothalamus and strong involvement of the brain stem, clearly distinguishable from BSE-L (which maintains its affinity towards cortices as seen in cattle field cases). Scrapie derived strain show a brain profile with cortical and hypothalamic tropisms. The *de novo* NZW strain shows a distinct tropism for the diencephalon (particularly hypothalamus) and brainstem while sparing the cortices.

**Fig 4:**
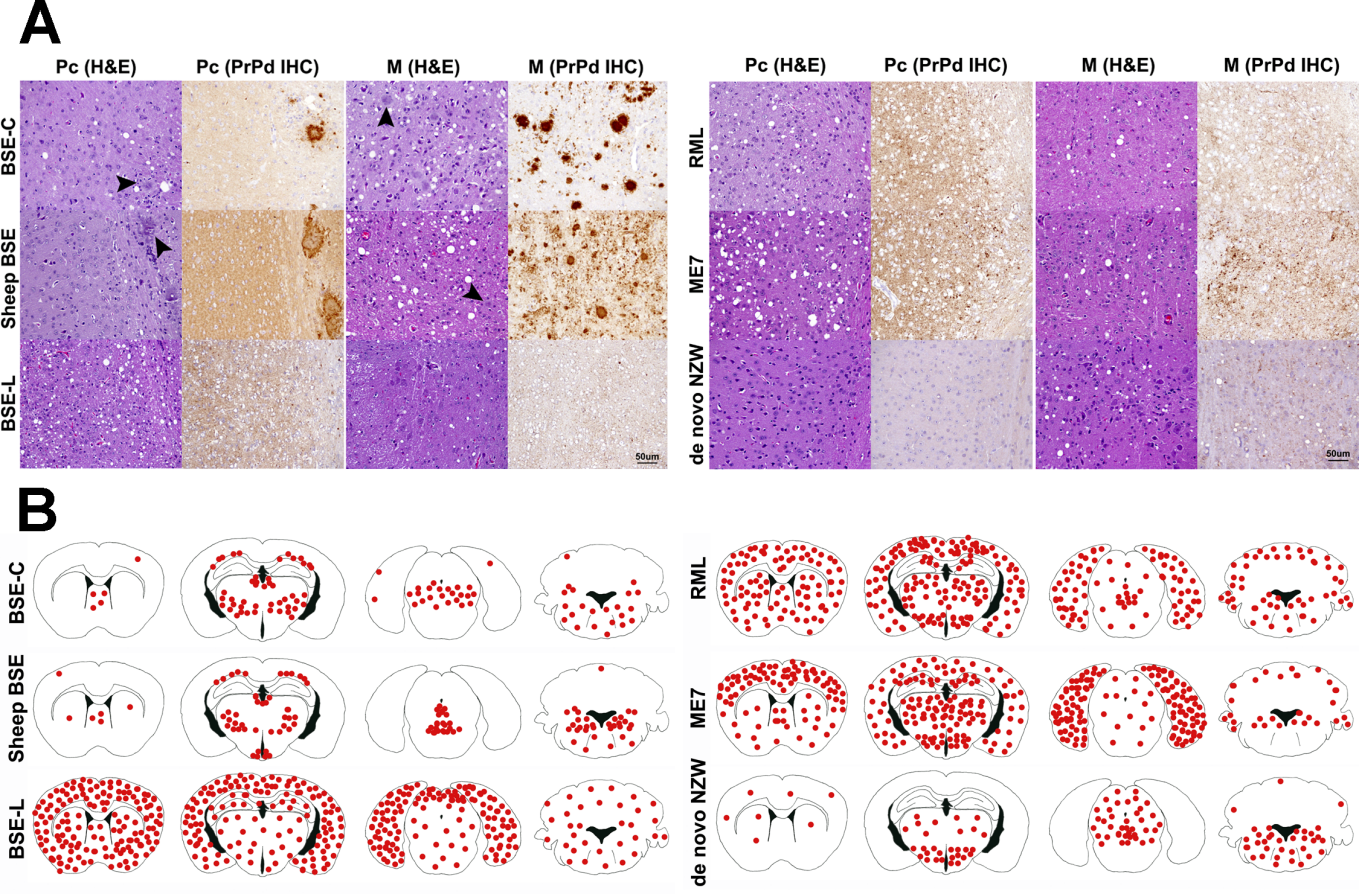
A: Histopathological characterization of several prion strains in *TgRab* mice. Classical BSE-derived strains show a remarkably similar lesion and PrP^d^ deposition patterns. PrP^d^ plaques are readily conspicuous in haematoxylin and eosin (H&E) stained sections (arrowheads). Upon immunohistochemical (IHC) staining with 6H4 antibody against prion protein, the predominant staining pattern consists of intensely stained round-shaped plaques which can coalesce and form large aggregates. Scrapie derived strains, on the other hand, as well as BSE-L and *de novo* NZW show a rather discreet, plaqueless, punctiform immunolabelling pattern found in the neuropil within and around neuronal bodies. Linear and stellate shaped PrP^d^ immunolabelling patterns are observed occasionally. In the case of RML, ME7 and BSE-L this pattern is also observed in the cortices while in *de novo* NZW the immunolabelling is restricted to the thalamus and brainstem. All images were taken at the same magnification. Bar 50 μm. B: Brain schematic mapping summary of the distribution of spongiform lesions and PrP^d^ deposits in the brains of *TgRab* mice. The red dots depict the areas where spongiosis and/or PrP^d^ deposits were mostly found in each group of infected mice. Note that the consistent picture yielded by BSE-C derived strains strongly involved the medulla oblongata, ventral mesencephalon, thalamus and deep parietal cortex while sparing the remaining cortices and the hypothalamus. This is clearly different from BSE-L which shows a remarkable tropism for the cerebral cortices and a lesser involvement of the brainstem structures. Scrapie derived strains, instead, show a clear tropism for the hypothalamus and a strong involvement of the cerebral cortices also. The spontaneous rabbit strain *de novo* NZW spares the cerebral cortices but shows a clear tropism for the hypothalamus.

Spongiform change and PrP^d^ distribution throughout the brain was semi-quantitatively assessed in histological sections of the inoculated brains of *TgRab* mice (Figs 3 and 4). Classical BSE-derived strains, namely BSE-C and sheep BSE, yielded very similarly shaped curves characterized by a strong involvement of the medulla oblongata, mesencephalon and thalamus but sparing of the hypothalamus. Involvement of the cortices and hippocampus was less intense but present, particularly at the deeper layers of the parietal cortex, involving the corpus callosum and sometimes extending to the oriens layer of the hippocampal formation. This pattern is equivalent to the one observed for BSE-C in the botg110 mouse model previously published by our group [36]. Mice inoculated with BSE-L, in contrast, showed a widespread involvement of the neocortex and less so in the diencephalon, mesencephalon and medulla oblongata in accordance with the brain PrP^d^ distribution observed in natural and experimental cases of BSE-L in cattle [18,38].

The type of PrP^d^ deposits seen by immunohistochemistry was also distinct in all mice inoculated with classical BSE-derived strains and consisted of amyloid-like rounded plaques, often confluent, which were readily visible on HE stained sections and positively stained in sections subjected to immunohistochemistry for PrP^d^ (Fig 4A). BSE-L inoculated mice lacked plaque type deposits and showed a very different punctate immunolabelling pattern in the neuropil and perikarya (Fig 4A). This was consistent with the pattern obtained in tgBov mice when inoculated with BSE-L.

The scrapie-derived strains RML and ME7 showed PrP^d^ deposits with a tropism for the diencephalon, including a consistent involvement of the hypothalamus (distinct from BSE strains), the mesencephalon and the medulla oblongata and also showed tropisms for the neocortex and cerebellar cortex. The PrP^d^ type, on immunohistochemistry, was distinguishable from that of BSE infected mice, as it was comprised of a fine punctate pattern in the neuropil and perikarya (Fig 4).

The lesion and PrP^d^ distribution of the rabbit-obtained *de novo* NZW strain showed a tropism confined to the diencephalon, including a consistent involvement of the hypothalamus, mesencephalon and medulla oblongata while sparing the cortices and hippocampus. The PrP^d^ type, on immunohistochemistry, consisted of a fine punctate pattern in the neuropil and perikarya resembling that observed in ME7 and RML infected mice.

### A model with spontaneous phenotype

The data presented validate the *TgRab* model to study rabbit susceptibility to prion strains. However, the *TgRab* line 058, chosen because it was the transgenic line showing the highest PrP^C^ expression levels, also showed a spontaneous phenotype secondary to PrP^C^ overexpression, as described by Westaway and coworkers [39], which needs to be taken into account when evaluating the results of any given experiment. Similar changes have been observed previously in other useful transgenic models overexpressing PrP^c^ [40,41]. In this phenotype, between 300 and 400 days, the majority (over 80%) of hemizygous mice (5x-6x PrP^c^ expression compared to normal levels; S2 Fig) developed gait abnormalities in the hindquarters that progressed slowly to complete hind-limb paralysis and atrophy of muscles (S3 Fig). The animals were able to feed, drink and groom normally and when it was not the case, as with any infected animal that reached the end point criteria, they were humanely euthanatized. See death time points for control groups in Fig 1. The same clinical presentation, but with enhanced severity, appeared in mice homozygous (10x-12x PrP^c^ expression levels) for the transgene as early as 60 days of age.

Microscopically, the skeletal muscle tissue showed irregular diameter of the muscle fibers along with the presence of anguloid fibers, centralization of nuclei and substitution by adipose tissue proliferation in the endomysium (S3E Fig and Fig 3). These changes are compatible with neurogenic atrophy. Lesions were observed also in the central nervous system and consisted of an intense spongiform change in the white matter, particularly in the corpus callosum and internal capsule (S4 Fig). The remaining brain parenchyma also showed diffuse moderate spongiosis, which was more evident in the diencephalon and brainstem and particularly intense in mice euthanized at older ages.

Even though no PrP^d^ was detected by western blotting or ELISA in any of the control animals, upon immunohistochemistry an intense PrP^C^ background immunolabelling was present throughout the brain in agreement with the known overexpression of PrP^C^. Additionally, a more intense labelling was observed, that could be mistaken for PrP^d^ signaling, which consisted of punctuate labelling around and within the cytoplasm of neurons, mainly located in the cortices but occasionally in the diencephalon and brain stem. Also, in the white matter, a punctate immunolabelling pattern was observed. Certain regions consistently showed strong immunolabelling of PrP^C^ including the cochlear nucleus in the medulla oblongata and the cerebellar cortex where a diffuse labelling was observed in the molecular layer and intense labelling in the granular layer, depicting the synaptic glomeruli (S4 Fig). Despite most of the animals displaying an overt overexpression phenotype, characterization allowed clear discrimination of this from *bona fide* prion infection in this model.

## Materials and Methods

### Inocula preparation for *in vivo* prion propagation studies

Brain homogenates (10^−1^ in PBS) for use as seeds for PMCA or direct intracerebral inocula were prepared manually using a glazed mortar and pestle from brains of animals clinically affected by various TSE: BSE-C and BSE-L field cases supplied by the Laboratorio Central de Veterinaria (Algete, Madrid, Spain), SSBP/1 and ME7 supplied by Animal Heath and Veterinary Laboratory Agency (New Haw, Addlestone, Surrey, UK), CWD from the thalamus area of the brain of a female Mule deer, genotype 225SS, infected with CWD (04–22412 WSV2 EJW/JEJ), supplied by Department of Veterinary Sciences (Laramie, WY, USA), RML supplied by Rocky Mountain Laboratories (Hamilton, MT, USA) and Sheep BSE supplied by Ecole Nationale Vétérinaire (Toulouse, France). The atypical scrapie isolate was obtained from a field case diagnosed in PRIOCAT laboratory, CReSA-IRTA (Barcelona, Spain). Rabbit spontaneous prions were those obtained in the rabbit bioassays conducted in the Moredun Research Institute, Scotland [23].

### Generation of *in vitro* PrP^res^ by serial automated PMCA (saPMCA)

The *in vitro* prion replication and PrP^res^ detection of amplified samples was performed as described previously with minor modifications [23,42]. Briefly, rabbit brains used for substrate were perfused using PBS + 5 mM EDTA and the blood-depleted brains were frozen immediately until required for preparing the 10% rabbit brain homogenates (PBS + NaCl 1% + 1% Triton X-100). 50–60 μl of 10% rabbit brain homogenate, either unseeded or seeded with the corresponding prion strain were loaded onto 0.2-ml PCR tubes and placed into a sonicating water bath at 37–38°C without shaking. Tubes were positioned on an adaptor placed on the plate holder of the sonicator (model S-700MPX, QSonica, Newtown, CT, USA) and subjected to incubation cycles of 30 min followed by a 20 s pulse of 150–220 watts sonication at 70–90% of amplitude. Serial rounds of PMCA consisted of 24-48h of standard PMCA followed by serial *in vitro* 1:10 passages in fresh 10% rabbit brain homogenate substrate. An equivalent number of unseeded (4–6 duplicates) tubes containing the corresponding brain substrate were subjected to the same number of rounds of saPMCA in order to control cross-contamination and/or the generation of spontaneous PrP^res^. The detailed protocol for PMCA, including reagents, solutions and troubleshooting, has been published elsewhere [43].

### Biochemical characterization of *in vitro*- and *in vivo*-generated prion strains

PMCA treated samples were incubated with 85–200 μg/ml of protease K (PK) for 1 h at 42°C with shaking (450 rpm) as described previously [44]. Digestion was stopped by adding electrophoresis Laemmli loading buffer and the samples were analyzed by Western blotting.

### Generation of *TgRab* mice

After isolation by PCR amplification using 5’ CCGCCGTACGTCATCATGGCGCACCTCGGCTAC 3’ and 5’ GGGGCCGGCCTCATCCCACGATCAGGAAG 3’ as primers, the open reading frame (ORF) of the rabbit PRNP gene was cloned into the pGEM-T vector (Promega). The rabbit-PrP ORF was excised from the cloning vector by using the restriction enzymes *BsiWI* (Thermo Fisher Scientific Inc.) and *FseI* (New England Biolabs Ltd.) and then inserted into a modified version of MoPrP.Xho vector [45] as described previously [46], which was also digested with *BstWI* and *FseI*. This vector contains the murine PrP promoter and exon-1, intron-1, exon-2 and 3’ untranslated sequences. The transgene was excised using *NotI* and purified with Invisorb Spin DNA Extraction Kit (Inviteck) according to the manufacturer recommendations.

Transgenic mouse founders were generated by microinjection of DNA into pronuclei following standard procedures [40]. DNA extracted from tail biopsies was analyzed by PCR using specific primers for the mouse exon 2 and 3’ untranslated sequences (5’ GAACTGAACCATTTCAACCGAG 3’ and 5’ AGAGCTACAGGTGGATAACC 3’). Those which tested positive were bred to mice null for the mouse Prnp gene in order to avoid endogenous expression of mouse prion protein. Absence of the mouse endogenous Prnp was assessed using the following primers: 5’ ATGGCGAACCTTGGCTACTGGC 3’ and 5’ GATTATGGGTACCCCCTCCTTGG 3’. The rabbit PrP expression levels of brain homogenates from transgenic mouse founders were determined by western blot using anti-PrP MAb L42 antibody (RIDA-Biopharm, Darmstadt, Germany) and compared with the PrP expression levels from NZW rabbit brain homogenates.

Animals homozygous for the transgene showed a spontaneous clinical phenotype as early as 60 days old, resembling the one described in the results section, but more severe, requiring euthanasia at 60–120 days old. Due to this, hemizygous mice were maintained for subsequent studies. The international code to identify this transgenic mouse line is STOCK-Prnp^tm2Edin^ Tg(moPrpn rabPrP)58Bps although throughout the paper they are referred to as *TgRab* mice.

### *TgRab* mice inoculation

Mice of 42–56 days of age were intracerebrally inoculated under gaseous anesthesia (Isoflurane) through the right parietal bone. A 50 μl SGC precision syringe was used with a 25 G gauge needle and coupled to a repeatability adaptor fixed at 20 μl. A dose of buprenorphine was subcutaneously injected before recovery to consciousness to reduce post-inoculation pain.

Mice were kept in a controlled environment at a room temperature of 22°C, 12 h light-darkness cycle and 60% relative humidity in HEPA filtered cages (both air inflow and extraction) in ventilated racks. The mice were fed *ad libitum*, observed daily and their clinical status assessed twice a week. The presence of ten different TSE-associated clinical signs [47] was scored.

The experimental groups are listed in Table 2. As the hemizygous mice had a slight spontaneous phenotype due to PrP^C^ overexpression (see Results), involving gait abnormalities, animals were euthanized following the end-point criteria (body weight, measurable clinical signs, physical appearance, unprovoked behavior and response to external stimuli). Positive TSE diagnosis relied principally on the detection of PrP^d^ (either by immunohistochemistry and/or western blotting or ELISA) and associated spongiform change in the brain parenchyma.

### Ethics statement

All experiments involving animals were approved by the animal experimentation ethics committee of the Autonomous University of Barcelona (Reference number: 585–3487) in agreement with Article 28, sections a), b), c) and d) of the “Real Decreto 214/1997 de 30 de Julio” and the European Directive 86/609/CEE and the European council Guidelines included in the European Convention for the Protection of Vertebrate Animals used for Experimental and Other Scientific Purposes.

### Sample processing and general procedures

When the clinical end-point criteria were reached, mice were euthanized by an overdose of pentobarbital administered intraperitoneally followed by decapitation. The brain was immediately extracted and placed into 10% phosphate buffered formalin. From each mouse a rostral section of the brain (including olfactory bulbs and frontal cortex), a caudal fraction of the medulla oblongata and the whole spinal cord were kept frozen (for biochemical studies and second passage). Transversal sections of the remaining brain tissue were performed at the level of the piriform cortex, optic chiasm and medulla oblongata. Samples were embedded in paraffin-wax after dehydration through increasing alcohol concentrations and xylene. Four micrometer sections were mounted on glass microscope slides which were stained with hematoxylin and eosin for morphological evaluation. Additional sections were mounted in 3-trietoxysilil-propilamine-coated glass microscope slides for immunohistochemistry.

### Detection of PrP^d^

A pool of all frozen central nervous tissues samples was homogenized 1:10 (W/V) in PBS using closed tubes containing ceramic beads, placed in a ribolyzer (Precess, Bio-Rad) and subsequently analyzed either by western blotting, as described above, or by ELISA (IDEXX, Herdcheck). The latter is a commercial ELISA based on the affinity of misfolded prions to an anionic substrate (termed Seprion). A new threshold was defined to adapt to the higher densitometry readings obtained when working with samples with PrP^c^ overexpression: only samples with a ratio spectrophotometry reading/cutoff over 5 were considered positive.

### Immunohistochemistry

Immunohistochemistry (IHC) against PrP^d^ was performed as described previously [48]. Briefly, deparaffinized sections were subjected to epitope unmasking treatments: immersed in formic acid and boiled at low pH (6.15) in a pressure cooker and pre-treated with proteinase K. Endogenous peroxidases were blocked by immersion in a 3% H_2_O_2_ in methanol. Then, the sections were incubated overnight with anti-PrP MAb 6H4 primary antibody (1:2000, Prionics AG) and subsequently visualised using the DAKO Goat anti-mouse EnVision system (Ref. K400111/0) and 3,3’diaminobenzidine as the chromagen substrate. As a background control, incubation with the primary antibody was omitted.

### Semi-quantification and data analysis

Histological lesions (i.e. spongiform change) and PrP^d^ immunolabelling were evaluated under a light microscope by a pathologist. A semi-quantitative approach was used to obtain comparable data from the different prions used to challenge mice. Spongiform lesion and PrP^d^ immunolabelling were scored separately. A total of 15 different brain regions were chosen: piriform cortex (Pfc), hippocampus (H), frontal cortex (Fc), parietal cortex (Pc), temporal cortex (Tc), occipital cortex (Oc), thalamus (T), hypothalamus (HT), mesencephalon (M), medulla oblongata (Mobl), cerebellar nuclei (Cm), cerebellar vermis (Cv) and cerebellar cortex (Cc). Scores ranging from (0) absence of spongiosis or immunolabelling: (1) mild, (2) moderate, (3) intense and (4) maximum intensity of lesion or immunolabelling were assigned to each brain area studied (Fig 3). Each area was investigated globally as region for the scoring. Brain profiles were plotted as a function of the anatomical areas which were ordered along the X axis in an attempt to represent the caudo-rostral axis of the brain. This methodology was adapted from a previous study performed on BSE-infected cattle [49]. Graphs were plotted using Microsoft Office 2007 Excel software.

## Discussion

This is the first report of *in vivo* evidence suggesting that *TgRab* mice are susceptible to cross species transmission of prion strains. This not only reinforces that rabbits can no longer be considered TSE resistant, but also that there is a possibility they could act as a reservoir for other prion strains. As such, rabbits must be taken into account when determining the epidemiology of several TSE both in relation to the species of origin, especially sympatric ones, but also to potential zoonotic transmission.

In previous studies we demonstrated that rabbits were able to propagate abnormal prions and that these were transmissible to other rabbits. However, this was only one prion strain which was generated *de novo* in an *in vitro* PMCA assay in rabbit brain homogenate (a spontaneous rabbit prion strain) and on first passage it had only a very limited attack rate [23]. This new mouse model, which responded in a comparable manner to rabbits when challenged with the same *in vitro* generated rabbit derived inoculum, has allowed us to evaluate the *TgRab*’s susceptibility to a number of actual field prions strains from a variety of different species. Although the use of rabbits would have been the most appropriate model there are strong, particularly budgetary, limitations due to the longer lifespan of rabbits and the need to use level 3 biosafety facilities. Thus, a transgenic mouse model overexpressing rabbit PrP^C^ was designed to overcome these limitations and allow us to determine its susceptibility to different prion strains.

No polymorphisms have been described in the PRNP rabbit gene, therefore several mouse transgenic lines were generated expressing rabbit PrP^C^ at different expression levels. The line with the highest possible PrP^C^ expression levels was selected to allow for easier prion propagation capacity but the overexpression was not so high as to generate a spontaneous phenotype at an early age which would preclude the attainment of infectivity/susceptibility data. The hemizygous *TgRab* line met these criteria with levels of PrP^C^ between 5 to 6 times those present in rabbits. The use of transgenic mice overexpressing ovine PrP^C^ to obtain the infectivity titer of specific prion isolates has been shown to be equivalent to titrations obtained through bioassay in the natural host [50]. Phenotyping of the newly developed prion transgenic model was essential, especially as the levels of PrP^c^ expression present have not been shown to be problematic in other models [41,46]. Eighty percent of the *TgRab* mice presented with a late onset spontaneous neurological disease phenotype (S3 Fig and S4 Fig) which, fortunately, did not interfere in the interpretation of prion susceptibility results. This allowed us to work with a model that faithfully reproduced the behavior in rabbits with respect to their capability to propagate different prion strains. One cannot exclude the possibility that the presence of spontaneous disease might create a toxic environment in the brain which artificially enhances the transmission of certain strains. Therefore a thorough knowledge of the PrP^C^ overexpression-related changes in uninfected controls was essential to identify the true prion disease status and validity of susceptibility.

Lesion morphology and profiling within the brain and identification of specific PrP^d^ deposition-types allowed unequivocal identification of infected animals (either spontaneous or as a result of an inoculation). Further biochemical detection of the presence of PrP^res^ by western blotting confirmed the ability of morphological techniques to identify an infected animal. Additionally, as PrP^C^ overexpression may mask an incipient infection, second passages are required to confirm if rabbits are totally resistant to those prion isolates to which they initially appeared to be, such as SSBP/1, atypical scrapie or CWD, and these experiments are ongoing.

Once validated the *TgRab* model was used to evaluate which TSE strains were able to infect the model (Table 2). Previous attempts in rabbits had concluded they were resistant, probably due to incomplete studies and the strong barrier of rabbits to propagate prions [34]. Initially classical cattle BSE, the most relevant field strain, was tested and found to be infectious on first passage with a low attack rate (4/9) and relatively long incubation period (551dpi±10). The strain properties observed in the infected *TgRab* mice (western blotting, brain lesion and PrP^d^ deposition profiles) were typical of BSE and indistinguishable from those obtained in other BSE murine models [36]. Parallel bioassay studies were conducted with the BSE isolate previously amplified *in vitro* using rabbit normal brain homogenate as a substrate (BSE-RaPrP^res^, this inoculum was characterised previously in a TgBov mouse model by our group [36]). These animals showed a 100% (12/12) attack rate and a shortened incubation period (396dpi ±12 vs 551dpi ±10) compared to the cattle BSE inoculated *TgRab* mice. This reduction already indicated that a transmission barrier between species had been overcome thanks to the *in vitro* adaptation of the cattle BSE-C to rabbit PrP^C^, a second passage was performed from that isolate which also showed a 100% attack rate (3/3). Its incubation period was reduced to 322dpi ±12 (mean ± s.e.m.) indicating further host adaptation (S5 Fig).

SheepBSE, derived from BSE-C, infected *TgRab* mice with a 100% attack rate (9/9), a relatively short incubation time (368±10 dpi) and with lesion and PrP^d^ brain profiles identical to those of BSE-C inoculated mice, suggesting that the same strain was being propagated through both isolates. This enhanced virulence of sheepBSE compared to BSE-C has been previously demonstrated in other experimental scenarios [29,51]. The results obtained with sheep scrapie differed completely as, in agreement with early experiments in rabbits [34], none of the *TgRab* mice inoculated with SSBP/1 showed any evidence of a prion disease on first passage. However, this result does not preclude that, if further *in vivo* SSBP/1 passages were to be performed, the transmission barrier would be crossed. As in the case of BSE in the bank vole (*Myodes glareolus*), where after an initial resistance a bank vole adapted BSE strain was obtained which was highly transmissible [52,53]. Conversely, ME7 and RML scrapie, both murine adapted sheep scrapie strains, infected *TgRab* mice on first passage and their incubation times, PrP^res^ biochemical profiles, lesion profiles and PrP^d^ deposition patterns were clearly distinguishable from cattle derived strains. Together these data are the first evidence that *TgRab* mice are not only able to propagate prions but they do it maintaining clearly the different distinguishing strain features (Figs 1, 3 and 4) which strongly suggests that rabbits may also.

It is noteworthy that both ME7 and RML, which originated from serial passages of SSBP/1 in different rodents [54,55], directly propagated in *TgRab* mice on first passage. Conversely, SSBP/1 did not infect *TgRab* mice on first passage. The murine adapted prion strains behaved differently to their parent strain, possibly because passage through rodents had selected for a strain capable of crossing the rodent species barriers. The situation is analogous to CWD which will infect hamsters after initial passage through ferrets [9]. In the present work, previous adaptation of scrapie to rodents, possibly resulting in a higher sequence identity in some specific and crucial PrP regions with rabbits compared to sheep, allowed rodent adapted scrapie prions to misfold rabbit PrP^C^. In previous studies ME7 did not infect rabbits after 4–5 years of incubation, with the exception of a single inconclusive case [23,34]. This result is difficult to extrapolate since we are discussing different species, of differing lifespans and with a species barrier between them. The PrP^C^ overexpression in *TgRab* may have allowed ME7 to propagate more efficiently than in rabbits which suggests that if the original rabbit experiments had been performed over the maximum lifespan of rabbits ME7 may have propagated on first passage also.

Once BSE in cattle has been virtually controlled, CWD in cervids is the animal prion disease with the most repercussions, at least in the North American continent. The uncertainty of its transmissibility to humans [56] and its unique ability to spread through the free ranging cervid population make its study highly relevant with respect to transmissibility to other species. Moreover CWD prions are known to be shed and are highly persistent in the environment. Rabbits are a sympatric species with cervids. Even though CWD has been shown to transmit on first passage to many species including sheep, cattle [57], squirrel monkeys [58], cats [59], hamsters [60], ferrets [9], mink [61], bank voles and deer mice (Genus *Peromyscus*) [62] its transmissibility efficiency is relatively low with very long incubation periods and low attack rates. For instance, wild type mice could not be readily infected, so tga20 mice overexpressing murine PrP^C^ were required to prove susceptibility to CWD [63] or required a second passage [64]. Another example is the transmission of CWD to cats, which required an incubation period of longer than 4 years [59]. The present study showed CWD was not able to infect *TgRab* on first passage (0/12). Further experiments are required to confirm the resistance of rabbits to CWD including a blind second passage and bioassays with CWD previously passaged in other species, especially rodents [9]. This will rule out an analogous situation as the one observed in this paper with sheep scrapie whereby SSBP/1 does not transmit to *TgRab* but murine passaged counterparts, ME7 and RML, do.

With respect to the atypical prion strains of purported spontaneous origin [18,65,66], BSE-L infected *TgRab* mice on first passage and, although the attack rate was low (3/11), they had the shortest incubation period observed in this model so far (221dpi for the first animal to die, mean 280±26dpi). The lesion and PrP^d^ deposition brain profiles differed considerably from those of BSE-C. None of the *TgRab* mice inoculated with atypical scrapie showed evidence of a TSE with the exception of one animal, euthanized at 742 dpi which, even though no histological lesions nor PrP^d^ deposits were present suggestive of infection, it was positive by PrP^d^ ELISA. This result could not be confirmed by western blotting. However, this ELISA detects PrP^d^ through its affinity to an anionic ligand not due to its resistance to protease K so we cannot rule out this single mouse was positive. A second passage is ongoing which will determine the result.

Initial *in vitro* experiments predicted that BSE as well as SSBP/1 and CWD isolates were able to missfold rabbit PrP^C^. However, a discrepancy was found with the bioassay results since neither SSBP/1 nor CWD infected *TgRab* mice on first passage. Several saPMCA rounds were needed in order to amplify the different isolates, varying in number depending of each strain. Thus, it is not surprising that on first passage some of the isolates do not transmit.

Besides the PRNP sequence, another component of the transmission barrier is the genetic background in which each PrP^C^ is contained. This has been demonstrated by infectivity studies showing BSE propagated more efficiently in RIII mice than C57/Black mice, two mice strains of the same species with the same PRNP gene [67]. Or when the genetic background (i.e. passage through different inbred mouse lines) determined not only the incubation period but also the propagation of two biochemically different BSE-derived strains [68]. For these reasons the belief that rabbits were resistant to prion infection was not only attributed to the rabbit PrP^C^ sequence but also to its genetic background. To study whether the genetic background of rabbits was responsible for the apparent prion resistance, Houdebine’s group generated transgenic rabbits expressing an ovine PrP^C^ which was known to easily misfold. Upon inoculation with ovine prion strains these rabbits succumbed to prion disease further proving that rabbits are not resistant to prions (results published paired with this article) and that the genetic background is not a limiting factor [37].

The differential susceptibility observed between actual rabbits and the transgenic model presented here can be explained by the higher PrP^C^ expression levels of *TgRab* mice. Lower expression mouse lines would probably only be susceptible on first passage to strains previously adapted to rabbit PrP^C^ as occurs with rabbits. It has taken more than three decades to finally dismiss the rabbit as a prion resistant species. We believe that the studies presented here confirm that *in vitro* studies are of great help in interpreting *in vivo* results, leave no room for misinterpretation, and that it can be ascertained that rabbits, and probably all other mammal species [21], are susceptible to infection by specific prion strains. The prion strain and its species of origin determine the extent of susceptibility, but neither rabbit PRNP nor their genetic background suggest they are resistant to prion propagation. Unfortunately, as with other mammals, the exact molecular mechanisms governing the capricious choice of strains that can be propagated in a certain species is still unknown.

In light of our results, especially susceptibility to spontaneous cattle prions (BSE-L), the restrictions on rabbits being fed ruminant protein should be maintained *sine die* to minimize the chances of any prion strain transmitting to rabbits.

## Supporting Information

S1 Fig Biochemical analysis of different rabbit PrP^res^ (RaPrP^res^) propagated *in vitro* by saPMCA using a NZW rabbit brain homogenate as substrate. Rabbit brain homogenates seeded with different prion strains (cattle: BSE-C, sheep: SSPB/1 and sheep BSE-C, deer: CWD) or unseeded (*de novo*) were subjected to saPMCA. Seeded samples from round 10 and the unseeded sample from round 20 from *in vivo* isolates were digested with 100 μg/ml of proteinase K (PK) and analyzed by western blot using monoclonal antibody 6H4 to compare their differential electrophoretic migration and glycosylation patterns. Control: Normal rabbit brain homogenate. MW: Molecular weight.
(TIF)

S2 Fig Biochemical analysis of undigested 10% (w/v) brain homogenates (*TgRab*, NZW rabbit and C57BL6 mouse) to evaluate the levels of PrP^C^ expression. Samples were diluted as described in figure and monoclonal antibody 6H4 was used at 1:10,000. The level of PrP^C^ expression observed in *TgRab* brain was 5–6 times higher than NZW rabbit brain and 10–12 times higher than mouse brain. MW: Molecular weight.
(TIF)

S3 Fig Characterization of the spontaneous phenotype observed in *TgRab*. **A and B**: Atrophy and paralysis of the hindquarters. **C**: Hindquarter footprints (black ink) of a healthy normal mouse. **D**: Footprints of a 500 days old non-inoculated mouse showing severe gait alteration. **E**: Histological image of the muscular tissue of a *TgRab* mouse with the spontaneous phenotype showing remarkable loss of muscular fibers (substituted by adipose tissue proliferation in the endomysium), irregular diameter and nuclear centralization in the remaining muscular fibers. **F**: normal muscular tissue of an unaffected mouse.
(TIF)

S4 Fig Histopathological characterization of the spontaneous phenotype observed in *TgRab* mice inoculated with normal brain homogenates of either healthy cattle, sheep or rabbit. All images were taken at the same magnification. Bar 50 μm. **A:** In some mice a severe spongiform change was observed in the corpus callosum (cc) and deeper layers of the neocotex, the image corresponds to parietal cortex (Pc). This lesion could also be observed in the internal capsule. **B:** Immunolabelling of PrP^d^ in the white matter showed punctiform morphology (arrowheads). **C:** Spongiosis of variable intensity was seen throughout the remaining brain areas which increased with the age of the mouse. In the image a section of thalamus with mild age-related spongiosis is seen. **D:** Parietal cortex. Punctifom immunolabelling around neurons was frequently observed in *TgRab* mice. Its distribution varied between mice but was present frequently in the cortices, striatum and thalamus. The fact that these cells are overexpressing rabbit PrP^C^ could be an explanation of this signaling. All these animals yielded a negative result to PrP^d^ by western blotting. **E:** The cochlear nucleus of the medulla oblongata consistently showed intense labelling of the neuropil in all negative controls. **F:** Cerebellar cortex. Intense, diffuse labelling of the neuropil was typically observed in the molecular (mol) and the granular (gra) layers and co-localized with the synaptic glomeruli.
(TIF)

S5 Fig Bioassay of *in vitro* amplified BSE-C using rabbit normal brain homogenate as a substrate (BSE-RaPrP^res^). **A:** *TgRab* survival times after inoculation. Each dot corresponds to a mouse. Dots with a red margin represent TSE positive animals. **B**: Brain lesion and PrP^d^ deposit distribution of the first passage of BSE-RaPrP^res^ in *TgRab* mice. **C**: 2^nd^ passage of BSE-RaPrP^res^ in *TgRab* mice. Lesion brain profiles and PrP^d^ deposition profiles represent the mean semi-quantitative scoring (0–4, vertical axis) of the spongiform lesions (black) and the immunohistochemical labelling of PrP^d^ deposits (red) against 14 brain regions (Pfc: piriform cortex, H: hippocampus, Oc: occipital cortex, Tc: temporal cortex, Pc: parietal cortex, Fc: frontal cortex, S: striatum, T: thalamus, HT: hypothalamus, M: mesencephalon, Mob: medulla oblongata, Cm: cerebellar nuclei, Cv: cerebellar vermis, Cc: cerebellar cortex). **D**: Histopathological characterization of BSE-RaPrP^res^ in *TgRab* mice. Lesion and PrP^d^ deposition patterns are remarkably similar to those observed in BSE C-derived strains. PrP^d^ plaques are readily conspicuous in haematoxylin and eosin (H&E) stained sections (arrowheads). Upon immunohistochemical (IHC) labelling with 6H4 antibody against prion protein, the predominant pattern also consists of intensely labelled round-shaped plaques which can coalesce and form large aggregates. All images were taken at the same magnification. Bar 50 μm. **E:** Brain schematic mapping summary of the distribution of spongiform lesions and PrP^d^ deposits in the brains of *TgRab* mice. The red dots depict the areas where spongiosis and/or PrP^d^ deposits were mostly found in each group of infected mice. The image is consistent with that found in BSE-C derived strains strongly involving the medulla oblongata, ventral mesencephalon, thalamus and deep parietal cortex while sparing the remaining cortices and the hypothalamus.
(TIF)
