# Supplementary figures and images for: Transgenic Mouse Bioassay: Evidence That Rabbits Are Susceptible to a Variety of Prion Isolates
Source: PLoS Pathog. 2015 Aug 6;11(8):e1004977. doi: 10.1371/journal.ppat.1004977 (PMC4527758; doi:10.1371/journal.ppat.1004977)

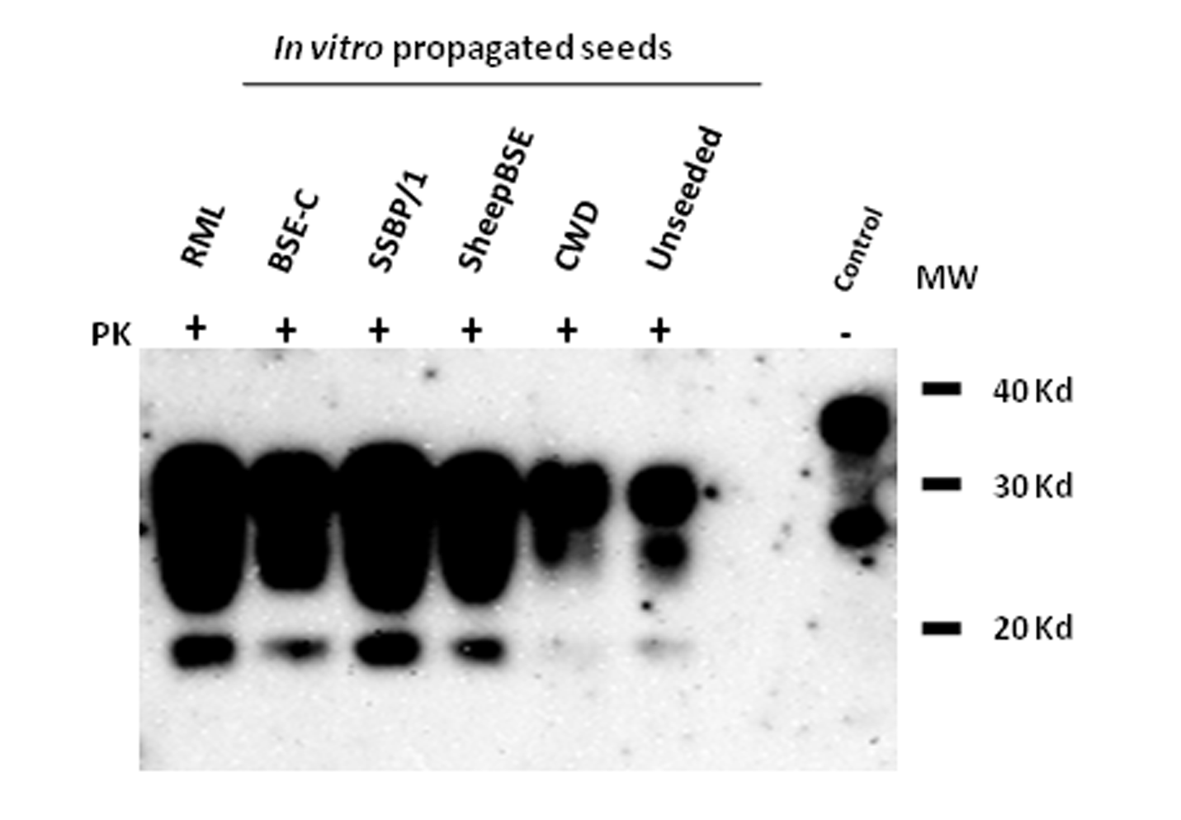

Supplement: S1 Fig — Rabbit brain homogenates seeded with different prion strains (cattle: BSE-C, sheep: SSPB/1 and sheep BSE-C, deer: CWD) or unseeded (de novo) were subjected to saPMCA. Seeded samples from round 10 and the unseeded sample from round 20 from in vivo isolates were digested with 100 μg/ml of proteinase K (PK) and analyzed by western blot using monoclonal antibody 6H4 to compare their differential electrophoretic migration and glycosylation patterns. Control: Normal rabbit brain homogenate. MW: Molecular weight. (TIF) [file ppat.1004977.s001.tif]

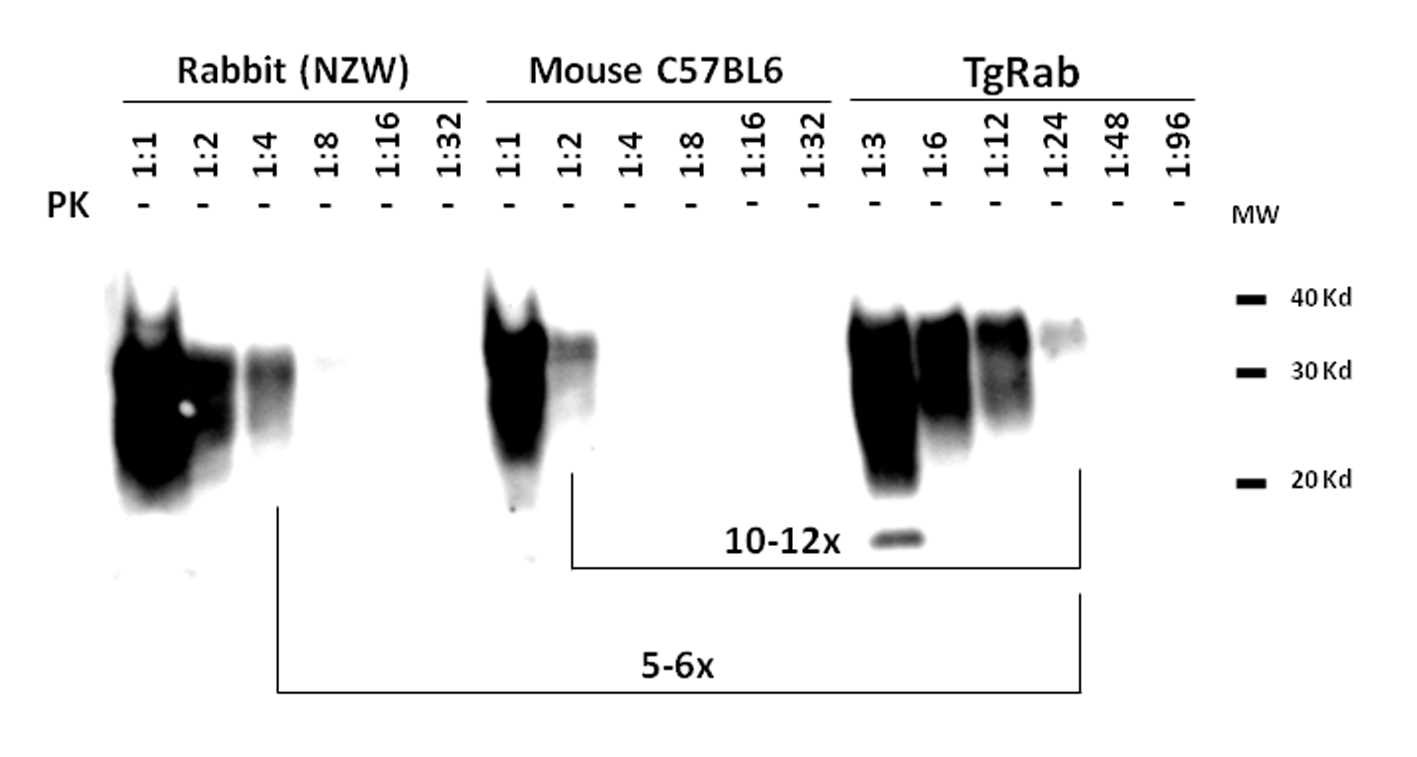

Supplement: S2 Fig — Samples were diluted as described in figure and monoclonal antibody 6H4 was used at 1:10,000. The level of PrPC expression observed in TgRab brain was 5–6 times higher than NZW rabbit brain and 10–12 times higher than mouse brain. MW: Molecular weight. (TIF) [file ppat.1004977.s002.tif]

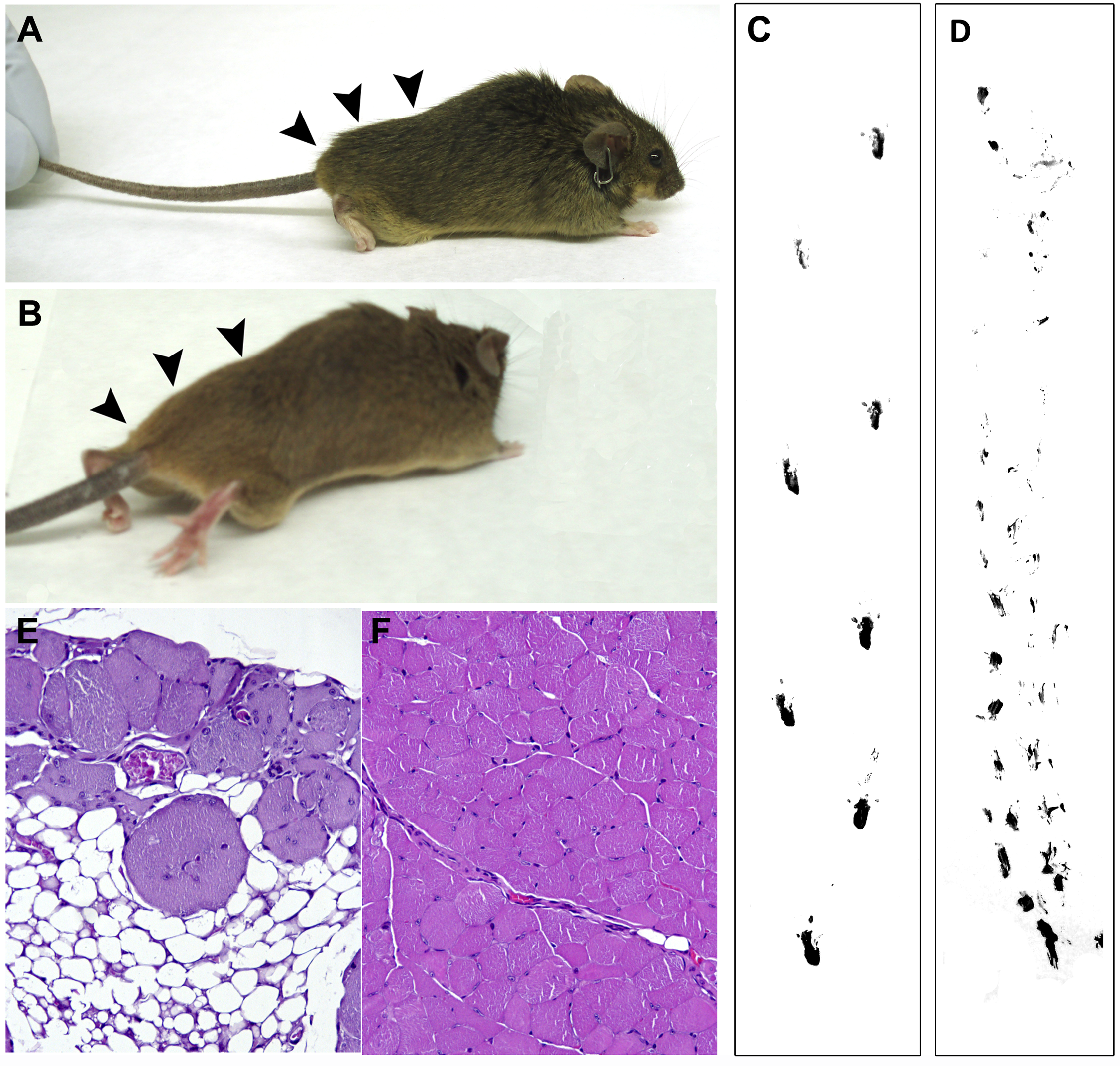

Supplement: S3 Fig — A and B: Atrophy and paralysis of the hindquarters. C: Hindquarter footprints (black ink) of a healthy normal mouse. D: Footprints of a 500 days old non-inoculated mouse showing severe gait alteration. E: Histological image of the muscular tissue of a TgRab mouse with the spontaneous phenotype showing remarkable loss of muscular fibers (substituted by adipose tissue proliferation in the endomysium), irregular diameter and nuclear centralization in the remaining muscular fibers. F: normal muscular tissue of an unaffected mouse. (TIF) [file ppat.1004977.s003.tif]

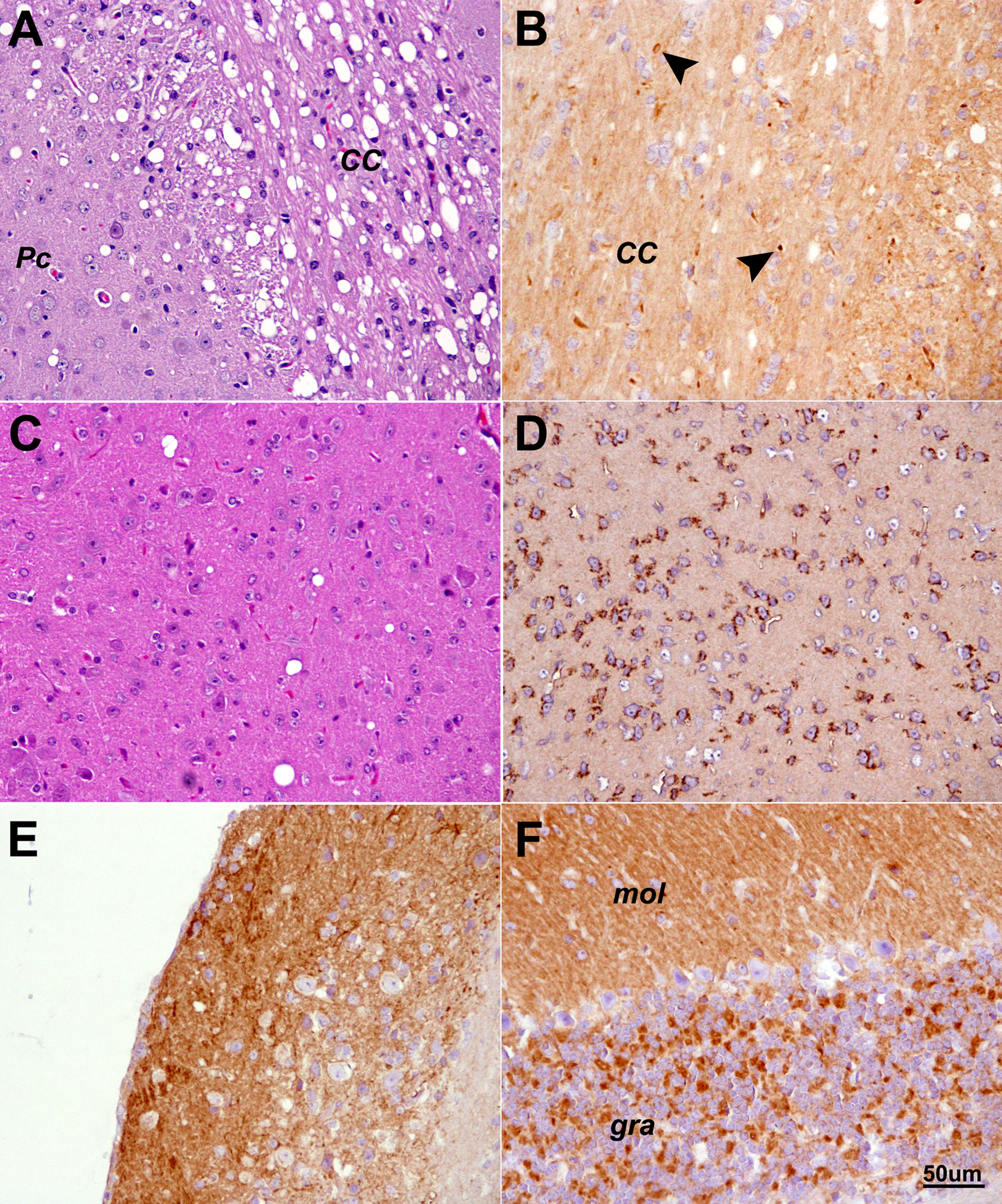

Supplement: S4 Fig — All images were taken at the same magnification. Bar 50 μm. A: In some mice a severe spongiform change was observed in the corpus callosum (cc) and deeper layers of the neocotex, the image corresponds to parietal cortex (Pc). This lesion could also be observed in the internal capsule. B: Immunolabelling of PrPd in the white matter showed punctiform morphology (arrowheads). C: Spongiosis of variable intensity was seen throughout the remaining brain areas which increased with the age of the mouse. In the image a section of thalamus with mild age-related spongiosis is seen. D: Parietal cortex. Punctifom immunolabelling around neurons was frequently observed in TgRab mice. Its distribution varied between mice but was present frequently in the cortices, striatum and thalamus. The fact that these cells are overexpressing rabbit PrPC could be an explanation of this signaling. All these animals yielded a negative result to PrPd by western blotting. E: The cochlear nucleus of the medulla oblongata consistently showed intense labelling of the neuropil in all negative controls. F: Cerebellar cortex. Intense, diffuse labelling of the neuropil was typically observed in the molecular (mol) and the granular (gra) layers and co-localized with the synaptic glomeruli. (TIF) [file ppat.1004977.s004.tif]

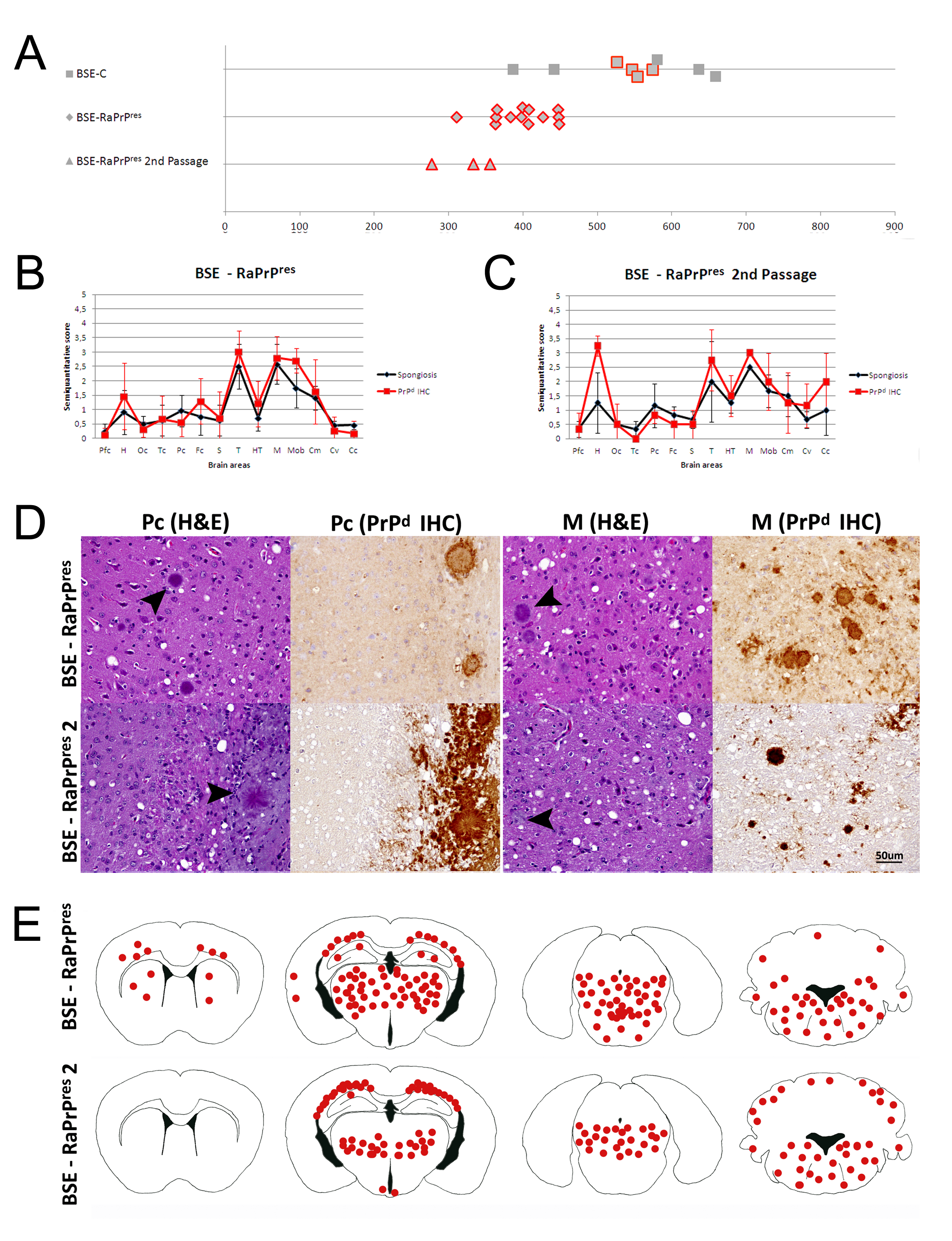

Supplement: S5 Fig — A: TgRab survival times after inoculation. Each dot corresponds to a mouse. Dots with a red margin represent TSE positive animals. B: Brain lesion and PrPd deposit distribution of the first passage of BSE-RaPrPres in TgRab mice. C: 2nd passage of BSE-RaPrPres in TgRab mice. Lesion brain profiles and PrPd deposition profiles represent the mean semi-quantitative scoring (0–4, vertical axis) of the spongiform lesions (black) and the immunohistochemical labelling of PrPd deposits (red) against 14 brain regions (Pfc: piriform cortex, H: hippocampus, Oc: occipital cortex, Tc: temporal cortex, Pc: parietal cortex, Fc: frontal cortex, S: striatum, T: thalamus, HT: hypothalamus, M: mesencephalon, Mob: medulla oblongata, Cm: cerebellar nuclei, Cv: cerebellar vermis, Cc: cerebellar cortex). D: Histopathological characterization of BSE-RaPrPres in TgRab mice. Lesion and PrPd deposition patterns are remarkably similar to those observed in BSE C-derived strains. PrPd plaques are readily conspicuous in haematoxylin and eosin (H&E) stained sections (arrowheads). Upon immunohistochemical (IHC) labelling with 6H4 antibody against prion protein, the predominant pattern also consists of intensely labelled round-shaped plaques which can coalesce and form large aggregates. All images were taken at the same magnification. Bar 50 μm. E: Brain schematic mapping summary of the distribution of spongiform lesions and PrPd deposits in the brains of TgRab mice. The red dots depict the areas where spongiosis and/or PrPd deposits were mostly found in each group of infected mice. The image is consistent with that found in BSE-C derived strains strongly involving the medulla oblongata, ventral mesencephalon, thalamus and deep parietal cortex while sparing the remaining cortices and the hypothalamus. (TIF) [file ppat.1004977.s005.tif]
